# A statistical shape model of the human sternum including cortical bone thickness

**DOI:** 10.3389/fbioe.2026.1872352

**Published:** 2026-08-07

**Authors:** Oscar Hallberg, Karl-Johan Larsson, Johan Davidsson, Anton Alexandersson, Anna Molnö, Shankharan Srinivasan, Johan Iraeus

**Affiliations:** 1 Department of Mechanical Engineering, Division of Vehicle Safety, Chalmers University of Technology, Gothenburg, Sweden; 2 Autoliv Research, Vårgårda, Sweden

**Keywords:** cortical bone thickness, demographic variation, PCA, statistical shape model, sternum morphology

## Abstract

**Introduction:**

Understanding population-level variation in bone size and shape is important for general biomechanics, clinical assessments, and for developing heterogeneous human body models used in safety restraint design. Thoracic injuries are common in severe motor vehicle crashes, and although rib fractures are frequently studied, sternal fractures also occur in these scenarios. This study aimed to investigate how demographic factors—sex, age, stature, and BMI—relate to variations in sternal size, shape, and cortical thickness, all important factors for fracture tolerance.

**Methods:**

Computed tomography scans from 56 female and 58 male subjects were segmented using a machine-learning-based pipeline to extract sternal geometries. A template mesh was fitted to each subject using landmarking and surface-fitting techniques, and cortical bone mapping was applied to estimate cortical thickness. Principal component analysis (PCA) was then used to quantify variation in shape and thickness. Multivariate linear regression models were created for each principal component (PC) using the demographic predictors and first-order interaction terms.

**Results:**

The leading principal components captured variation in cortical thickness distribution, overall size, the manubriosternal joint, and the positions of the sternocostal joints. There were significant demographic trends for five PCs, which explained 39.4% of the total variance in the PCA. When using the significant regression trends to predict sternal shape and cortical thickness in the original sample, *R* ^2^-values were 0.22 for spatial coordinates and 0.07 for cortical thickness, indicating substantial unexplained individual variation. The mean sternum had an average cortical thickness of 0.92 mm.

**Discussion:**

Predictor-isolation analyses indicated that sex exerted a stronger influence on overall size than stature. Further, sternal width increased with age, and individuals with higher BMI tended to exhibit slightly greater cortical thickness. While regression models were developed to explore demographic associations, the predictive performance was limited, indicating that the model should primarily be interpreted as a descriptive representation of population-level variation rather than a predictive tool.

## Introduction

In 2021, an estimated 1.2 million people died in road traffic crashes (World Health Organization, 2023). Considering the Abbreviated Injury Scale (AIS) (Association For The Advancement Of Automotive Medicine et al., 2015), thoracic injuries are the most frequent AIS3+ (serious) injuries sustained in frontal motor vehicle crashes (MVCs) (Carter et al., 2014; Forman et al., 2019; Weaver et al., 2015). When AIS2 injuries are included, the thorax becomes the most commonly injured body region among individuals over 65 years old (Östling et al., 2024). Fatality risk from thoracic trauma increases further in older populations compared to younger ones (Battle et al., 2023; Bansal et al., 2011; Forman et al., 2019; Carter et al., 2014; Brumbelow, 2019). Rib fractures are the most common thoracic injury, but sternal fractures are also prevalent, accounting for 31.7% of all chest wall injuries among patients requiring healthcare in one study (Benhamed et al., 2022). Some studies even report that sternal fractures occur more frequently than rib fractures in frontal MVCs (Pipkorn et al., 2020) and that the presence of rib fractures alone does not reliably predict the presence of sternal fractures (Ranmal et al., 2024). Across MVCs resulting in hospitalization, sternal fractures are reported in approximately 4% of patients (Brookes et al., 1993; Yeh et al., 2014; Khoriati et al., 2013), and their presence is considered an indicator of severe thoracic trauma (Güler and Koznali, 2021). Compared to other blunt chest trauma, frontal collision leads more often to sternal flail, especially when bilateral rib fractures are present (Caragounis et al., 2021). Sternal fractures are classified as moderate (AIS2) injuries. Despite this moderate AIS score, sternal fractures remain important to study for a comprehensive understanding of thoracic injury patterns, especially when considering the injury severity score (ISS) that combines the severity of several injuries in trauma patients.

In adults, the sternum consists of three bones: the manubrium, the sternal body, and the xiphoid process, connected by the manubriosternal and xiphisternal joints. These symphyseal joints are cartilaginous and allow limited movement, although they may be partially or completely ossified (Ehara, 2010; Sarcinelli et al., 2019). While all three components form the anterior thoracic wall, their clinical relevance in blunt trauma differs substantially. In a study of 108 cases, 60% of sternal fractures occurred in the manubrium and 40% in the sternal body, whereas no fractures were observed in the xiphoid process (Şimşek et al., 2022). Because the xiphoid process contributes minimally to fracture patterns and exhibits high anatomical variability, including absence (Vatzia et al., 2021), it was excluded from further morphometric analysis due to the difficulty of establishing consistent correspondence across subjects.

To support vehicle restraint development, human body models (HBMs) are used to evaluate restraint system performance. These models can be based on 3D data from a single individual or on population-level morphometric models (statistical shape models) that describe how the size and shape of a body part correlate with demographic predictors such as sex, age, stature, or BMI (Hwang et al., 2016). Morphometric models have been developed for several body regions, including the pelvis (Brynskog et al., 2021), ribs (Holcombe et al., 2024), femur (Klein et al., 2015), and ribcage (Shi et al., 2014). For the sternum, (Weaver et al., 2014), developed a statistical shape model (SSM) describing age-related changes in size and shape from 0 to 100 years for males and females. However, this model did not include stature or BMI, factors previously shown to correlate with the shape and size of other bones (Brynskog et al., 2021; Holcombe et al., 2024). Additionally, the manubrium and sternal body were treated as a single structure, preventing analysis of variation in the manubriosternal joint gap, an anatomically and mechanically relevant feature, as joint stiffness may influence sternal response (Kerrigan et al., 2010). Finally, cortical bone thickness was not examined, despite its known influence on fracture risk predictions in other flat bones (Larsson et al., 2023), suggesting it may also be important for sternal fracture risk.

Therefore, the aim of this study was to characterize variation in sternal morphology and cortical thickness and to examine their associations with sex, age, stature, and BMI. The study treats the manubrium and sternal body as separate bones to capture variability, also in the manubriosternal joint gap. Developing a detailed morphometric sternal model represents a natural step toward a more comprehensive understanding of thoracic variation within the population.

## Materials and methods

**FIGURE 1:**
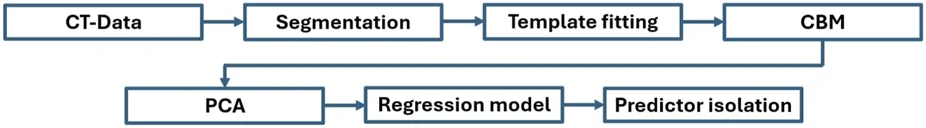
Schematic view of the methodology.

Computed Tomography (CT) image data were segmented using a machine-learning-based pipeline. A template mesh was then morphed to fit each subject’s sternum using landmarks and surface fitting techniques. Cortical bone thickness was estimated for each subject using cortical bone mapping (CBM). Next, shape and thickness variability were analyzed using principal component analysis (PCA). Finally, multivariate linear regression models were developed to correlate bone variations with demographic predictors. The overall methodology is shown in Figure 1, and the detailed steps are described in the following sections.

### Data description

This study used data from the New Mexico Decedent Image Database (NMDID), which contains full-body CT scans of non-embalmed cadavers in the supine position, along with associated demographic information (Edgar et al., 2020). The cadavers in the database were scanned after passing away due to sudden circumstances, and not due to a long-term injury or illness. The CT scans have an in-plane resolution ranging from 0.675 × 0.675 mm to 1.375 × 1.375 mm, and a slice thickness of 0.5 mm. The adult sample used in the present study includes subjects with ages ranging from 18 to 95 years old, statures ranging from 145 to 195 cm, and BMIs ranging from 15 to 45 kg/m^2^. These values approximately span the 5^th^ percentile female to the 95^th^ percentile male in the adult American population (Fryar et al., 2021). Subjects with a history of sternotomy were excluded, as were sternums containing a foramen (i.e., a hole in the bone), a feature present in approximately 10% of individuals (Babinski et al., 2015).

### Segmentation

Segmentations of the manubrium and sternal body were generated from the CT scans using a deep learning–based algorithm built on an nnU-Net architecture (Alexandersson and Molnö, 2025). The nnU-Net architecture was selected because it has demonstrated strong performance in medical image analysis tasks, such as segmentation, for limited data sets (Isensee et al., 2019). To train the model, ten manually segmented sternums were created using the software 3D Slicer 5.10 (Kikinis et al., 2014). Each sternum produced by the model was subsequently inspected manually for accuracy. Approximately 20% of the segmentations were discarded due to inaccuracies, primarily involving misclassification between the manubrium and the sternal body (i.e., the algorithm incorrectly assigning regions of the manubrium as sternal body and *vice versa*).

### Landmarking and template mesh fitting

**FIGURE 2:**
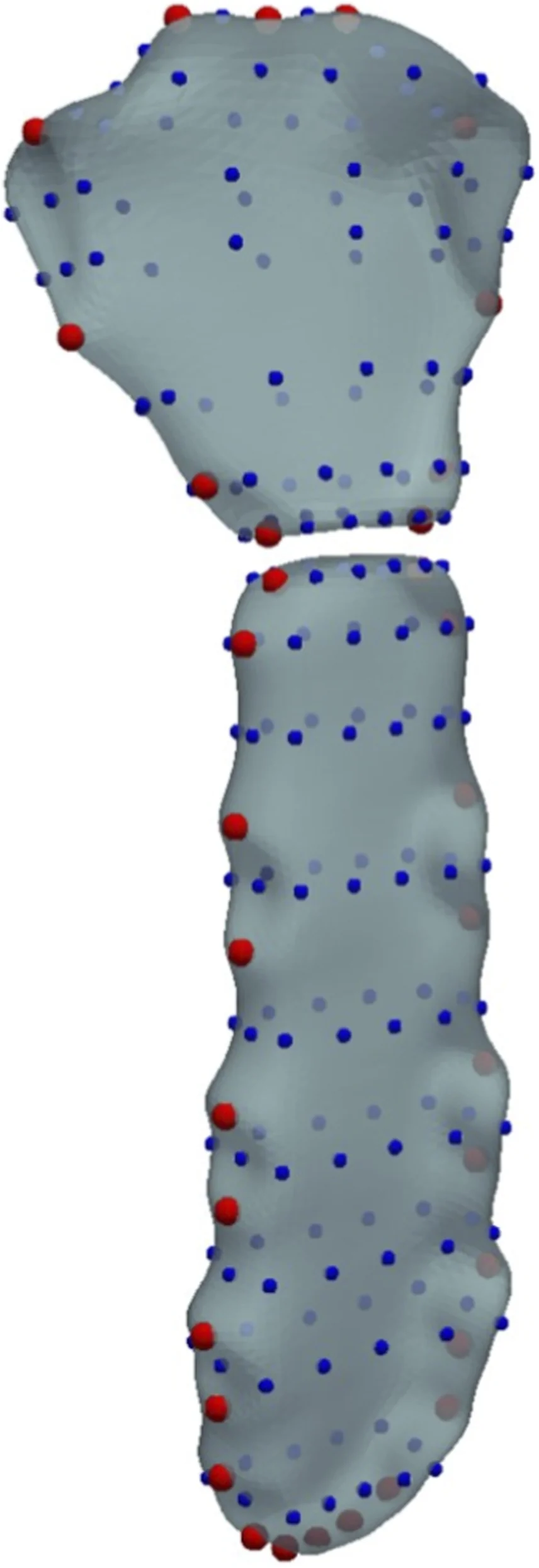
Landmark pattern on the manubrium and the sternal body. Red dots represent manually selected homologous landmarks, and blue dots represent pseudo-landmarks.

First, a landmark pattern consisting of 212 landmarks was placed at prominent anatomical features. 34 of these were manually placed homologous landmarks, while the remaining landmarks were pseudo-landmarks positioned equidistantly between the homologous ones. The number of landmarks was selected empirically based on visual inspection to ensure that all relevant anatomical features of the sternum were adequately represented and that the surface was uniformly covered by landmarks. The landmark configuration (Figure 2) was designed to capture overall sternal morphology of the manubrium and sternal body, while also capturing local features such as the grooves of the sternocostal joints and the thickness of the manubriosternal joint gap. Although formal interobserver testing of landmark placement was not performed, the landmark protocol relied primarily on clearly identifiable anatomical structures.

Next, a template mesh, with 18,764 vertices, was fitted to the shape of each individual sternum in a three-step process to create detailed and consistent shape descriptions. First, the 212 landmarks were used in a radial basis function (RBF) interpolation–based step to create an initial nodal alignment (Hu et al., 2016). This was followed by orthogonal projection of the morphed nodes onto the segmented surface, and a final in-plane smoothing step that applied a controlled, mild smoothing to achieve a uniform mesh density and to remove undesired sharp edges. This procedure produced a surface-matched representation of each subject using a consistent mesh topology.

### Extracting cortical bone thickness

The sternum segmentations were used together with the corresponding CT scans to estimate cortical bone thickness using CBM. Specifically, the CBM v2 algorithm (Treece et al., 2010), implemented in Stradview 7.4 (Graham Treece; Machine Intelligence Laboratory, Department of Engineering, University of Cambridge) with the settings detailed in Supplementary Appendix A, was applied. This method was selected because it has demonstrated good accuracy for relatively thin cortices (Treece and Gee, 2015). For each subject, the estimated thickness values were mapped onto the surface-fitted template meshes from the previous step, resulting in an estimated cortical thickness value at each node.

### Principal component analysis

General Procrustes Analysis was applied to the surface-fitted meshes to align all subjects into a shared reference system, thereby removing variations caused by differences in patient positioning during the CT scan.

To compute principal components (PCs) describing both spatial position and cortical thickness within the same analysis, nodal coordinates and nodal cortical thickness values were centered and normalized separately. This was done by first subtracting the mean of all nodal x-, y-, and z-coordinates, and the mean of all nodal thickness values, respectively, across all sternums. Next, the spatial coordinates were scaled by dividing them by the standard deviation of the $L_{2}$ -norm of all nodal coordinates, where the $L_{2}$ -norm for node i in sternum j is calculated as in Equation 1.

$$L_{2ji}=\sqrt{x_{ji}^{2}+y_{ji}^{2}+z_{ji}^{2}}$$ (1)

Similarly, the cortical thickness values were divided by the standard deviation of all nodal thickness values across all sternums. PCA was then performed on a concatenated matrix containing both the nodal coordinates and cortical thickness values of the subject-specific template meshes.

### Linear regression model

To predict sternum shape and cortical thickness, multivariate linear regression models were created to estimate PC scores from demographic variables. The covariates included age, sex, stature, and BMI, along with all first-order interaction terms. Separate regression models were fitted for each PC. Model performance was evaluated using 10-fold cross-validation. All possible combinations of main effects and interaction terms were assessed using the Akaike Information Criterion (AIC), and the combination with the lowest AIC was selected. To ensure model hierarchy, main effects were required to be present whenever their corresponding interaction terms were included. To control the family-wise error rate arising from multiple model evaluations, a Bonferroni correction was applied (Dunn, 1961). Specifically, the nominal significance level $\left(\alpha =0.05\right)$ was divided by the total number of predictor combinations $\left(n=112\right)$ , resulting in an adjusted significance threshold according to Equation 2.

$$\alpha_{Bonferroni}=\frac{0.05}{112}\approx 0.0004$$ (2)

If the p-value for the selected predictor combination was <0.0004, the model was considered significant and retained in the full regression model. All PCs with a significant regression model were subsequently used to predict sternum shape and cortical thickness based on demographic variables.

To evaluate the accuracy of the morphometric model, sternums for all subjects in the dataset were predicted by applying each subject’s demographic data to the regression models. Coefficient of determination ( $R^{2}$ ) was computed separately for spatial coordinates and cortical thickness as:

$$R_{coords}^{2}=1-\frac{\sum {\left(y_{ji}-\hat{y_{ji}}\right)}^{2}}{\sum {\left(y_{ji}-\bar{y_{i}}\right)}^{2}}$$ (3)

$$R_{thick}^{2}=1-\frac{\sum {\left(t_{ji}-\hat{t_{ji}}\right)}^{2}}{\sum {\left(t_{ji}-\bar{t_{i}}\right)}^{2}}$$ (4)

where $y_{ji}$ are the predicted coordinates for a specific sternum j , $\hat{y_{ji}}$ are true coordinates of that specimen, and $\bar{y_{i}}$ are the coordinates for the average sternum. The notation for cortical thickness, t -variables in Equation 4, follows the same pattern as the spatial coordinates in Equation 3. Given the relatively small sample size and the inclusion of multiple interaction terms, there is a risk of overfitting. The results of the regression analysis should therefore be interpreted in an exploratory rather than confirmatory context.

### Exploring the effect of demographic predictors

Next, the regression model was used to predict the average male and average female sternum. Demographic reference values were taken from (Schneider et al., 1983), resulting in an average female aged 45 years, with a stature of 161.8 cm and a BMI of 23.7, and an average male aged 45 years, with a stature of 175.3 cm and a BMI of 25.1. To further explore how individual demographic variables contribute to shape and cortical thickness variation within the studied population, selected predictors were varied in isolation. This approach was used to examine the effect of sex, as well as the effects of age, BMI, and stature separately for males and females. For each analysis, the non-varied predictors were fixed at the average values for the respective sex, while the isolated predictor was varied across a reasonable range, as presented in the Results section.

## Results

**FIGURE 3:**
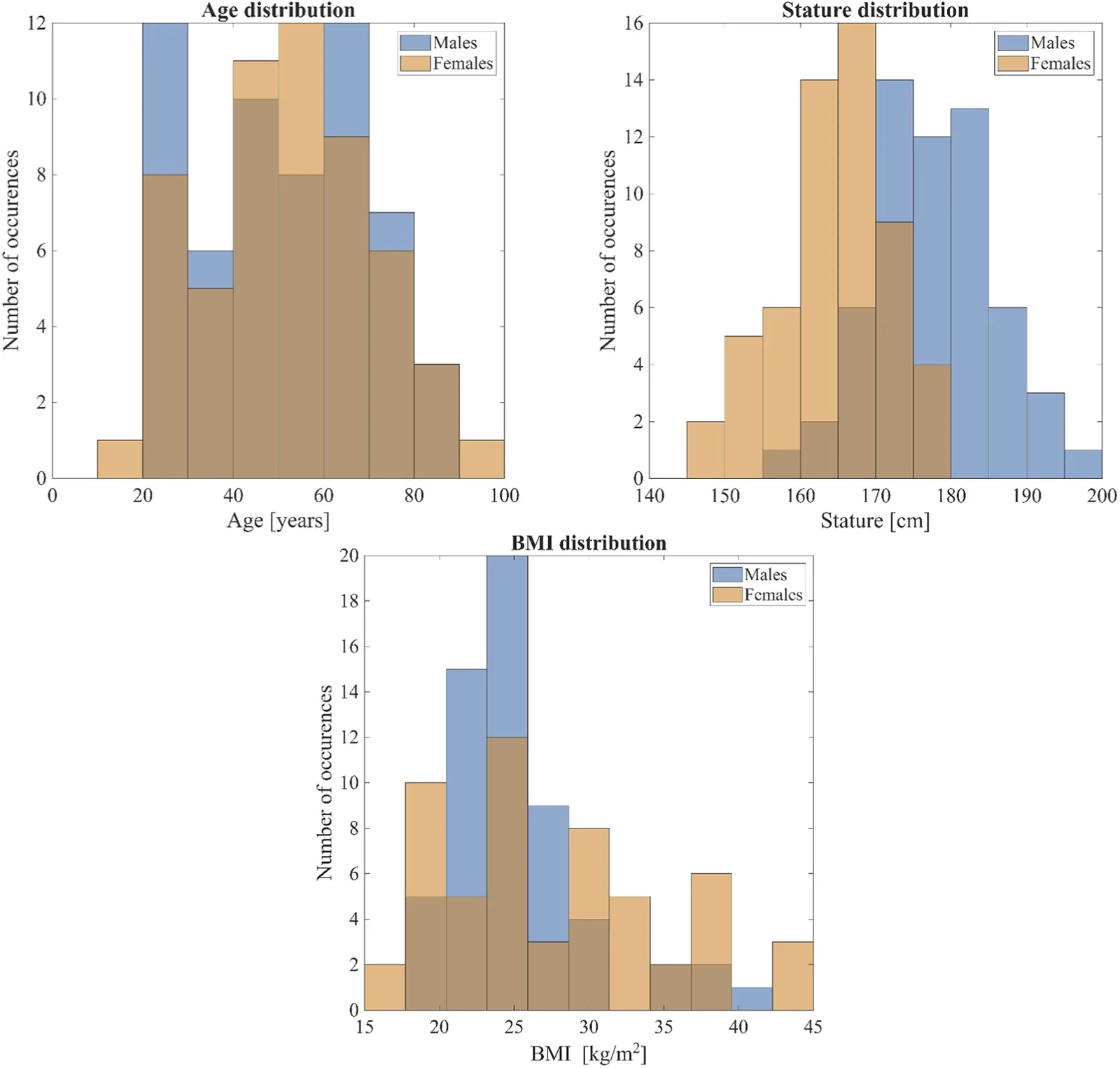
Age, Stature, and BMI distribution for the dataset. Brown areas represent the overlap between males and females.

The final dataset consisted of a total of 114 subjects (56 females and 58 males). The distribution of age, stature, and BMI for the included individuals is shown in Figure 3.

### Mean shape and cortical thickness

**FIGURE 4:**
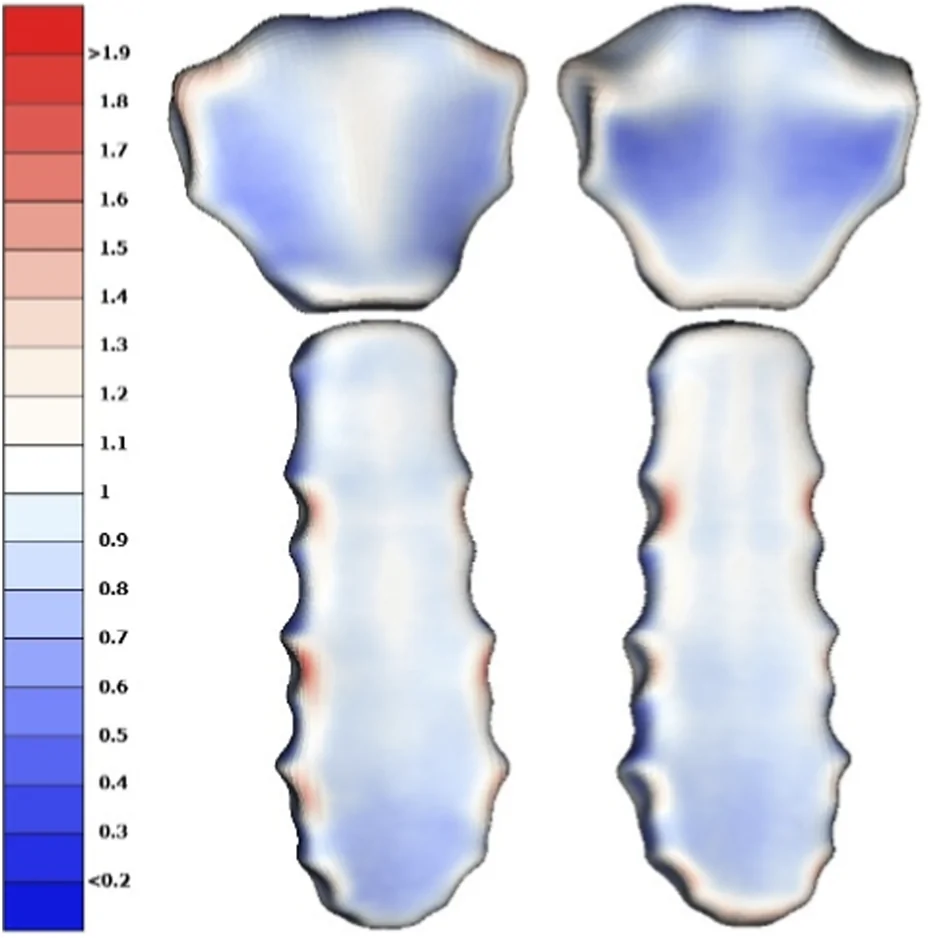
Average manubrium and sternal body shape with cortical thickness values [mm] represented using a colormap.

The average shape and cortical thickness distribution of the manubrium and sternal body are shown in Figure 4. This mean sternum represents the combined average of both male and female subjects. The average sternum length was 147.9 mm, the manubriosternal joint angle measured 164.9°, and the mean cortical thickness across the surface was 0.92 mm.

### Principal component analysis

**FIGURE 5:**
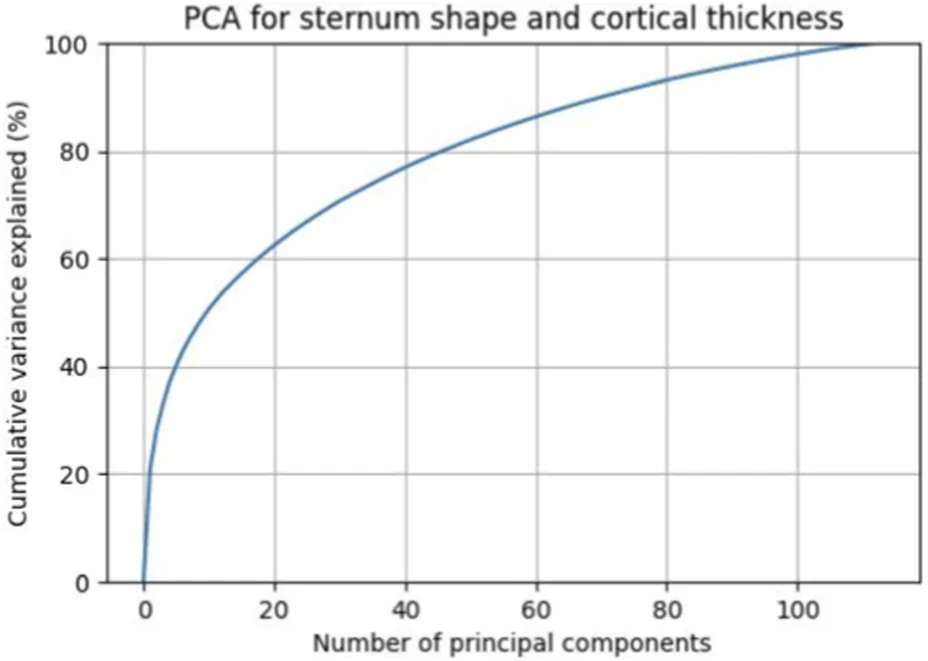
Cumulative explained variance from the principal components for sternal shape and cortical thickness.

The variance explained by the principal components (PCs) is presented in Figure 5, where the cumulative variance is plotted across all components.

The effects of the first five PCs on both shape and cortical thickness are illustrated at ±2 standard deviations (SD) from the mean PC scores in Table 1. Thickness deviations are shown in millimeters relative to the mean sternum thickness. Because each PC captures global variation, multiple shape and thickness changes occur simultaneously. However, the first PCs, which explain a larger proportion of the variance, exhibit more distinct and interpretable patterns. For example, PC1 primarily reflects variation in cortical thickness distribution, whereas PC2 mainly captures overall size differences.

**TABLE 1:** Visual and descriptive summary of the first five principal components.

PC Number	Anterior side		Posterior side	
Explained variance				
Description	−2 SD	+2 SD	−2 SD	+2 SD
PC1 20.82% Mainly cortical thickness variation; also, variation in manubriosternal joint position and inferior width	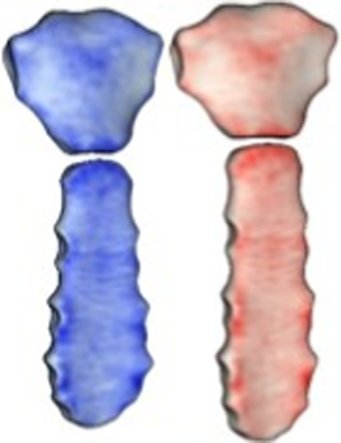		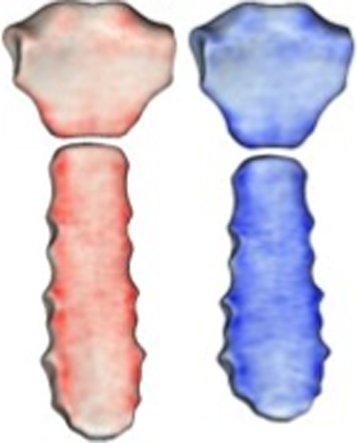	
PC2 7.43% Mainly overall sternum size; also, manubrium shape and manubriosternal joint position	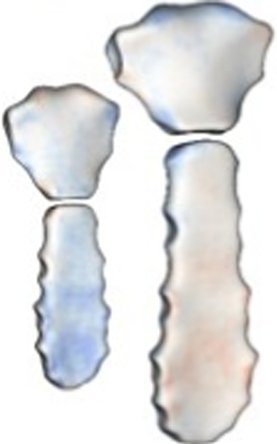		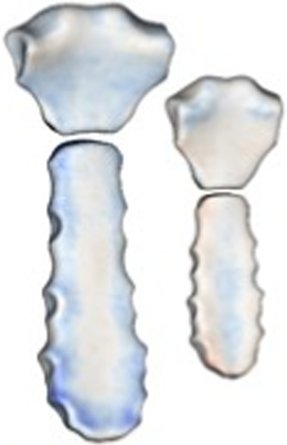	
PC3 4.77% Mainly variation in the manubriosternal joint gap; also, sternal body length, and the angle between the manubrium and the sternal body	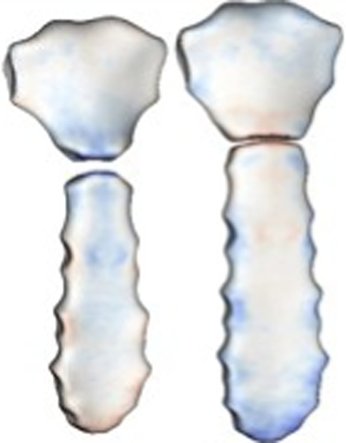		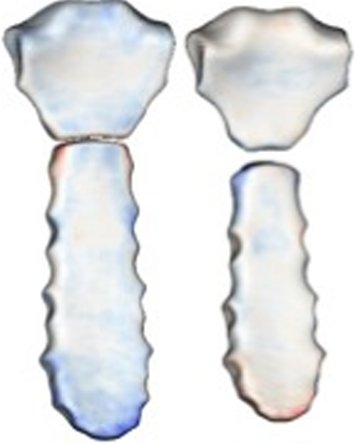	
PC4 4.12% Mainly sternum width; also, lateral and anterior/posterior cortical thickness variation	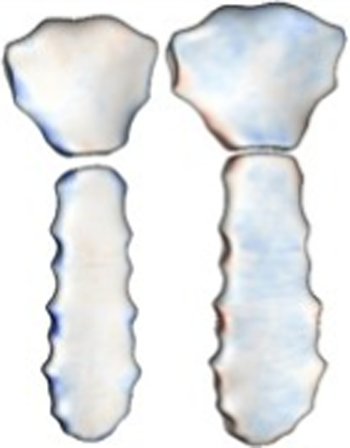		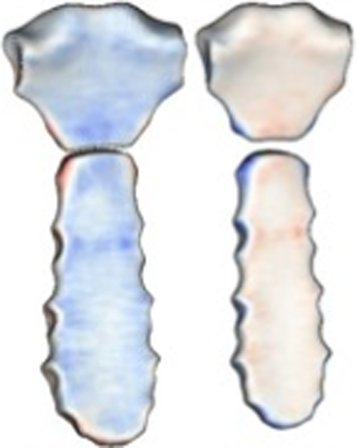	
PC5 3.02% Mainly sternal body shape; also, cortical thickness variation at the 4th and 5th costal cartilage attachments	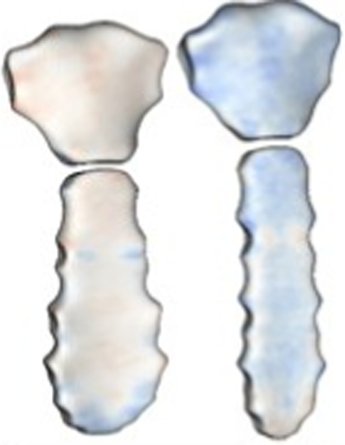		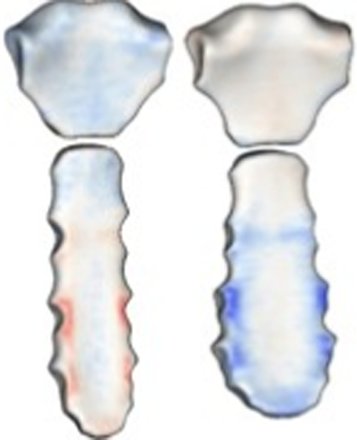	
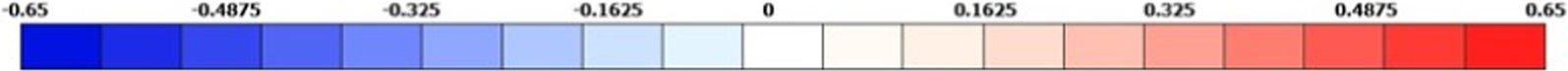				

Colors represent deviations (in mm) from the average sternum at each node, corresponding to ±2 SD of each PC.

### Regression model

Only PC1, PC2, PC3, PC4, and PC7 showed significant associations with the demographic predictors, and together they explained 39.4% of the overall variation from the PCA. The PCs included in the regression model, along with their corresponding predictors, are listed in Supplementary Appendix B. The predictive performance of the morphometric model resulted in $R_{coords}^{2}=0.22$ for spatial coordinates and $R_{thick}^{2}=0.07$ for cortical thickness.

### Predicting the average male and female

**FIGURE 6:**
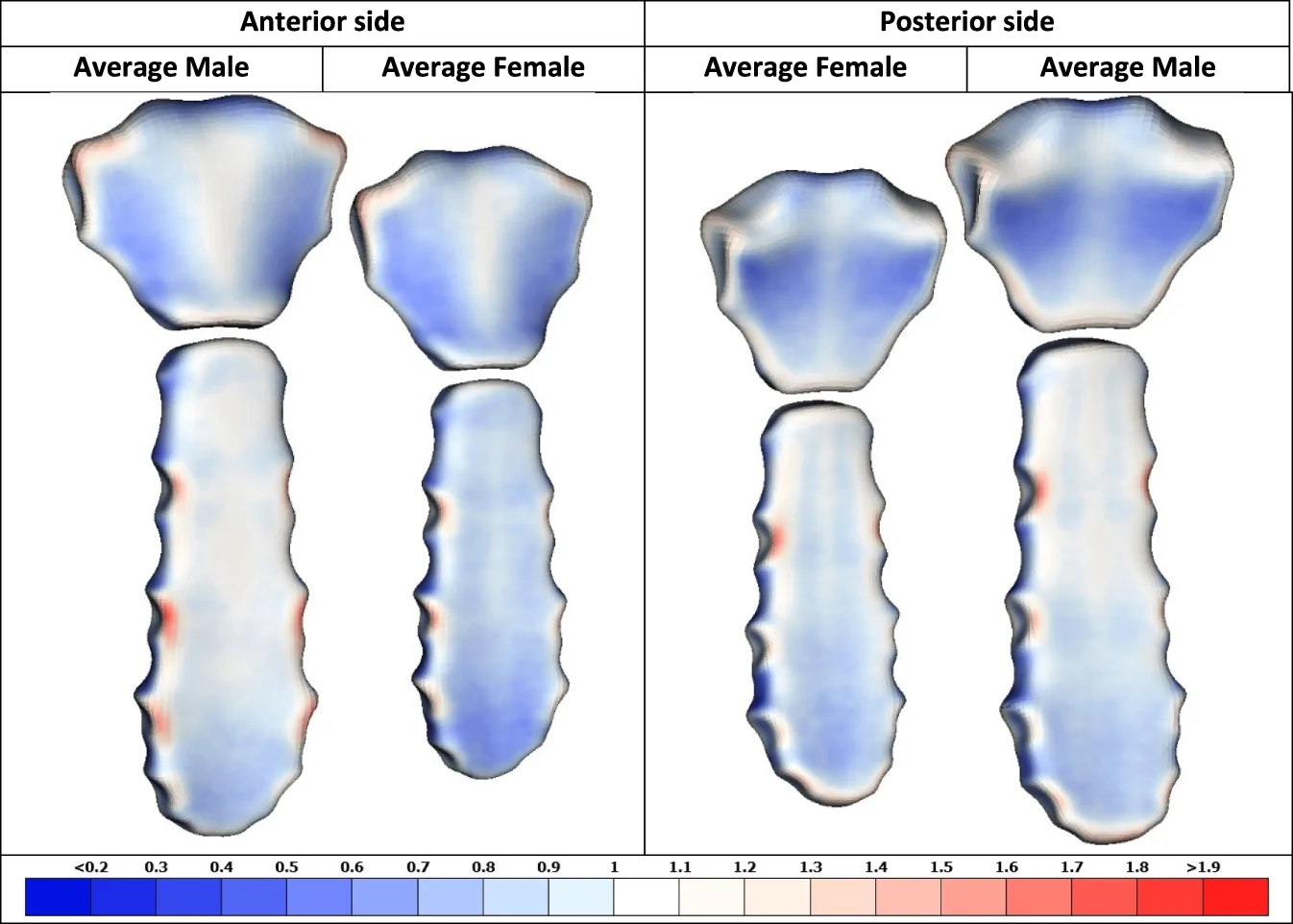
Predicted average male and female sternum. The colors represent cortical thickness in millimeters.

The predicted average male and female sternums are shown in Figure 6. Overall, the average male sternum was larger and exhibited greater cortical thickness compared to the average female sternum. The predicted mean cortical thickness was 0.88 mm for females and 0.94 mm for males, and the total sternum length was 135.0 mm for females and 158.5 mm for males.

### Predictor isolation

**FIGURE 7:**
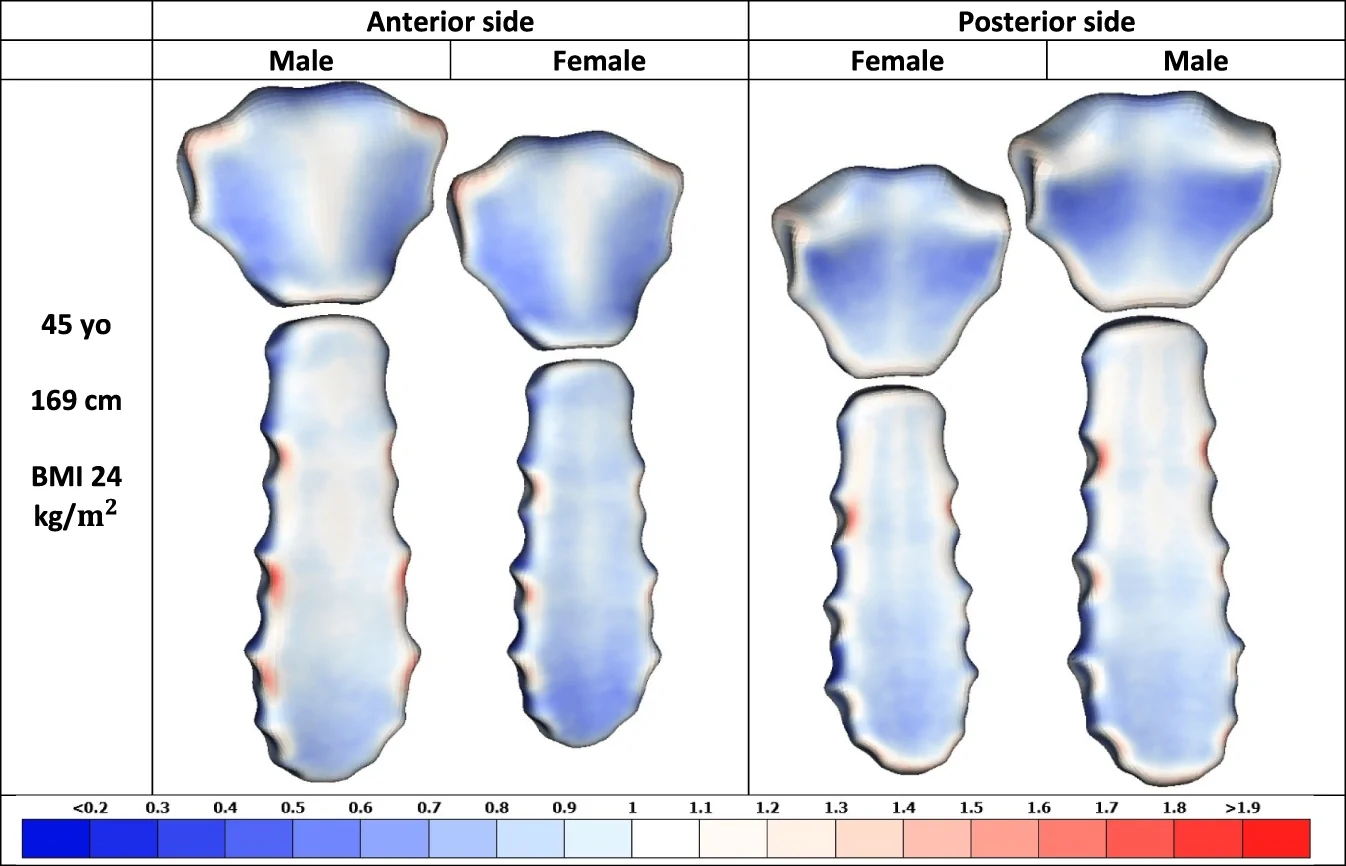
Comparison of how sex influences sternal shape and cortical thickness. The colors represent cortical thickness in millimeters.

**FIGURE 8:**
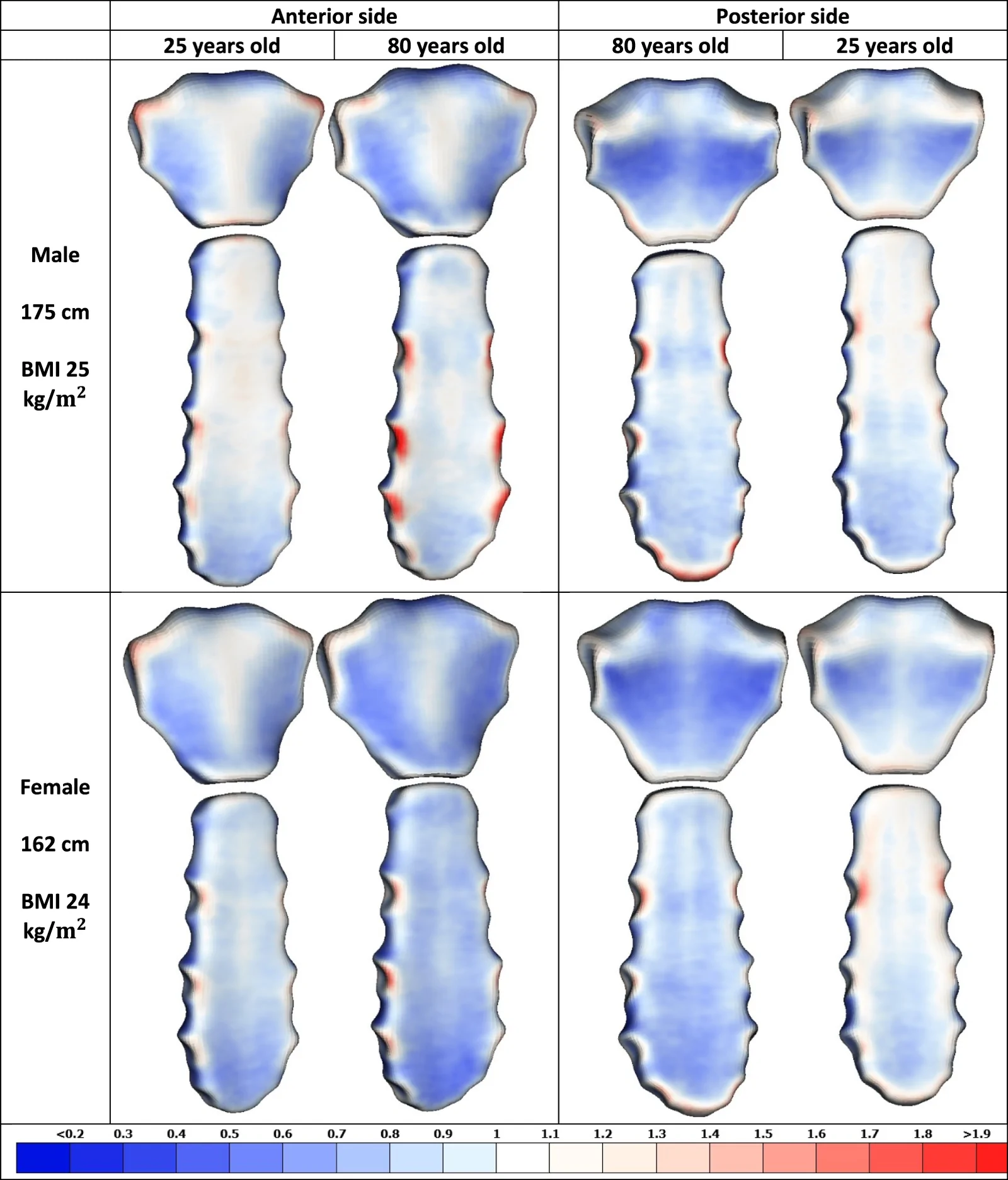
Comparison of how age influences sternal shape and cortical thickness for males and females. The colors represent cortical thickness in millimeters.

**FIGURE 9:**
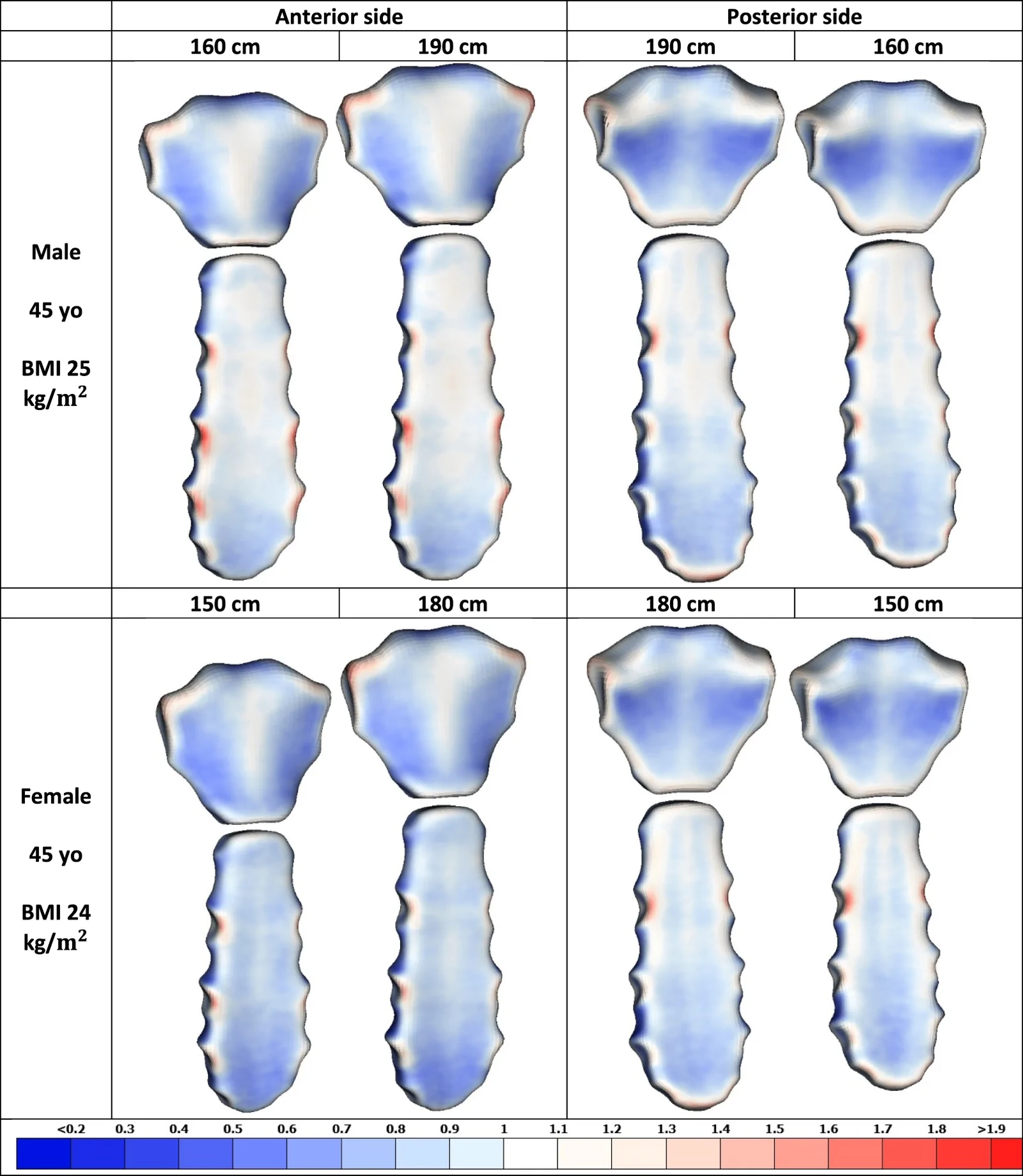
Comparison of how stature influences sternal shape and cortical thickness for males and females. The colors represent cortical thickness in millimeters.

**FIGURE 10:**
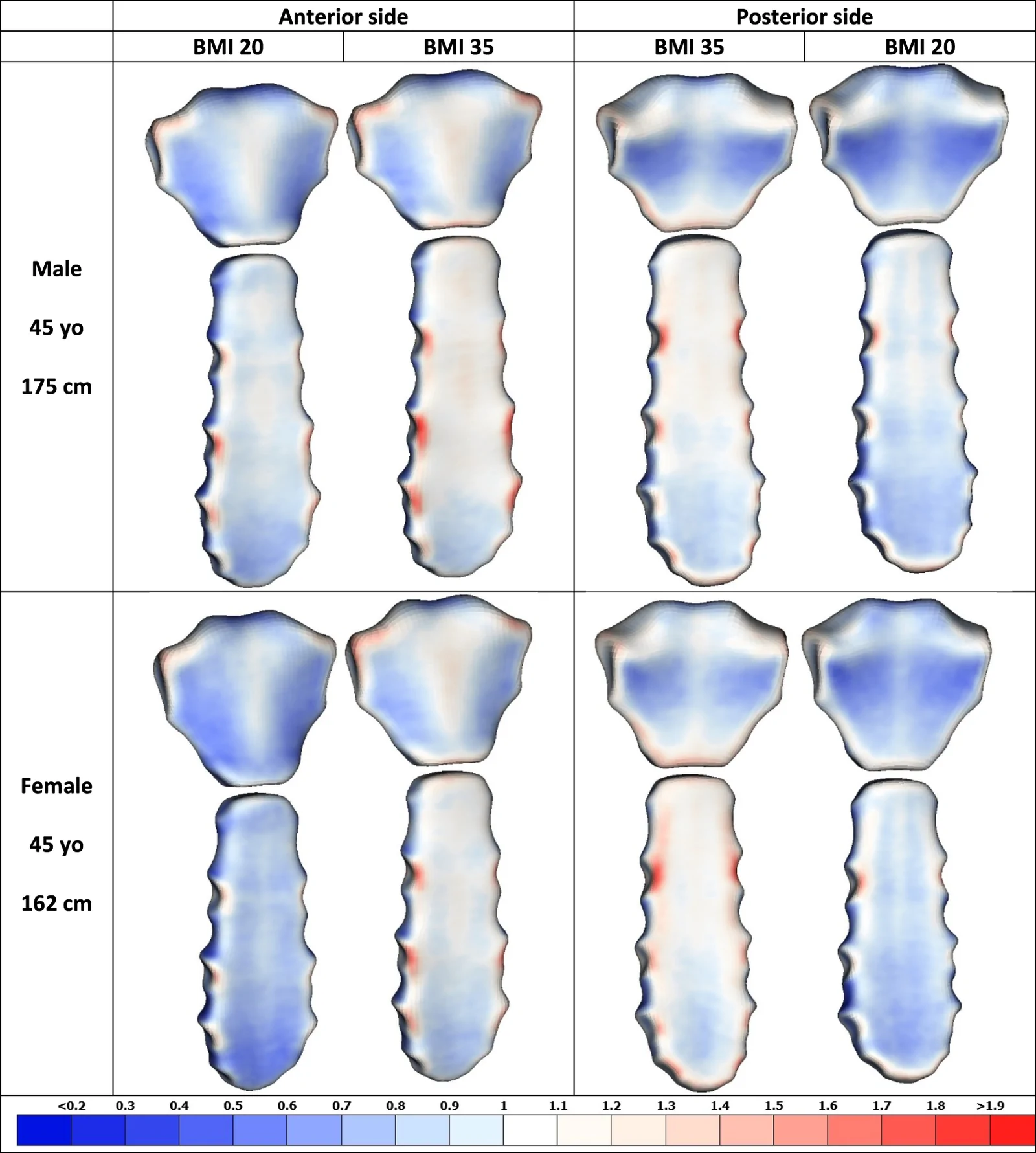
Comparison of how BMI influences sternal shape and cortical thickness for males and females. The colors represent cortical thickness in millimeters.

The results from the predictor isolation analyses are presented in Figures 7–10. The isolated sex effect shows that male sternums are generally larger than female sternums (at the same stature, BMI, and age), while female sternums exhibit a higher manubrium proportion of the total sternum length (Figure 7). The age effect indicates that cortical thickness decreases with increasing age, accompanied by an increase in inferior sternal width (Figure 8). Stature primarily influences overall sternum length, although the effect is less pronounced than the sex effect (Figure 9). Finally, the BMI effect demonstrates that individuals with higher BMI tend to have a thicker sternal cortex (Figure 10).

## Discussion

This study developed a comprehensive morphometric model of the human manubrium and sternal body that integrates size, shape, and cortical thickness. By combining a dense template-based mesh, detailed landmarking, and cortical bone mapping, the model captures both global morphology and fine-scale anatomical features, including the sternocostal grooves and the manubriosternal joint gap. In doing so, it extends previously published sternal morphometric models by incorporating cortical thickness variation alongside external shape.

The regression framework further enabled the identification of demographic trends in sternal morphology, demonstrating how factors such as sex, age, stature, and BMI contribute to variation in both shape and cortical thickness. These population-level trends provide a foundation for future studies aiming to identify sternal characteristics associated with increased vulnerability, for example, in clinical populations or in injury biomechanics. Moreover, the model offers a valuable resource for improving human body models, particularly in the context of predicting sternal fracture risk in MVCs, where both geometry and cortical thickness influence the structural response.

### Comparing results with literature

The present morphometric model can be compared with the sternum model developed by (Weaver et al., 2014). The most notable difference is the higher resolution of the sternocostal joints in the current study, likely due to the consistent identification of these features during the landmarking and surface-fitting process. In contrast, the lateral surfaces in the Weaver model appear smoother and lack the distinct sternocostal groove pattern captured here. Another methodological difference is that Weaver treated the sternum as a single bone, without separating the manubrium and sternal body, which prevented their model from representing the manubriosternal joint. Direct comparison of absolute dimensions is difficult because Weaver et al. included both children and adults.

The predicted average male and female sternums from the current model can also be compared with previous studies reporting sex-specific sternal measurements. (Selthofer et al., 2006). measured 35 female and 55 male sternums, and (Öner et al., 2018) reported sternal body and manubrium lengths from 30 males and 30 females. A comparison of these measurements with the present predictions is shown in Table 2. Except for the maximum manubrium width and the manubrium length in the Selthofer study, the average sternum dimensions in the current study are within the reported data variability, although the sternums in the current study tend to be slightly smaller. Because neither Selthofer nor Öner reported stature, age, or BMI, it is uncertain whether their samples represent a similar demographic subpopulation as the one used for the predictions in this study.

**TABLE 2:** Comparison of the average male and female predictions with (Selthofer et al., 2006; Öner et al., 2018).

Metrics [mm]	Female			Male		
	Selthofer ( ± 1 SD)	Öner (min – Max)	Current study – Average female	Selthofer ( ± 1 SD)	Öner (min – Max)	Current study – Average male
Manubrium maximum width	60.6 ± 8.1	-	51.7	68.2 ± 8.0	-	59.2
Manubrium minimum width	31.3 ± 3.3	-	29.1	36.8 ± 5.5	-	34.7
Manubrium length	52.4 ± 4.5	47.6 (42.1–56.7)	47.1	55.2 ± 3.6	53.6 (43.2–62.7)	49.0
Manubrium avg thickness	11.2 ± 0.9	-	11.1	12.6 ± 1.9	-	12.9
Body avg width	27.1 ± 3.1	-	27.0	30.7 ± 4.3	-	30.8
Body length	94.2 ± 14.0	87.5 (68.4–109.9)	85.4	109.7 ± 14.4	101.1 (84.1–131.6)	106.5
Body avg thickness	9.2 ± 1.2	-	8.9	10.0 ± 1.1	-	9.9
Sternal angle [°]	165.3 ± 7.2	-	163.5	166.4 ±7.4	-	163.6

Two additional studies report sternal metrics without distinguishing between sexes. First, (Lindner et al., 2017), report a manubrium length of 51 mm and a sternal body length of 98 mm, which closely match the average sternum derived from the PCA in the present study (48 mm and 97 mm, respectively). Second, (Bunn et al., 2019), reported a manubriosternal joint angle of 163.7°, which is close to the value of 164.9° found here. Overall, the predicted sternal dimensions in this study align well with previously published measurements. To the authors’ knowledge, no study exists that reports sternal cortical thickness, making the present work the first to report population-level cortical thickness distributions of the sternum.

### Segmentations

Since a new segmentation algorithm was used in the study, the segmentation performance was evaluated with Dice coefficients to assess the accuracy. The Dice coefficient quantifies the overlap between predicted and manually annotated ground-truth segmentations on a scale from 0 to 1, where values above 0.9 are generally considered excellent for anatomical structures (Seghier, 2024). The segmentations achieved scores of 0.964 for the manubrium and 0.954 for the sternal body during cross-validation (Alexandersson and Molnö, 2025). To further assess segmentation performance and to evaluate the interobserver concordance, a comparison between the segmentations produced by the nnU-Net model and a human rater was performed for one representative subject segmented by a human. The manual segmentation achieved Dice coefficients of 0.989 and 0.991 for the manubrium and sternal body, respectively, while the automatic segmentation achieved 0.982 and 0.966. These results indicate that the automatic method performed on par with manual segmentation methods and captured the relevant anatomical boundaries with high fidelity. Despite the strong performance, segmentation accuracy cannot be guaranteed in all cases. Distinguishing between adjacent anatomical structures in CT images remains challenging, particularly in regions with thin cortices where the contrast between bone and surrounding soft tissue is limited. Such uncertainties may have introduced small local deviations in the extracted surfaces and, consequently, in the cortical thickness estimates. However, the high Dice scores and the close agreement between manual and automatic segmentations suggest that these uncertainties were minor relative to the overall morphological variation captured in the model.

### Cortical bone mapping

Cortical bone mapping in this study was performed using the CBM v2 algorithm implemented in Stradview (Treece et al., 2010). The method has previously been validated for femoral CT images with a resolution of 0.33×0.33×1 mm (Treece and Gee, 2015), demonstrating good accuracy for both thick cortical regions in the femoral shaft and thinner cortices near the metaphysis. However, the method has not been explicitly validated for the sternum, as no ground-truth cortical thickness measurements were available for this study. Based on the femur validation, the algorithm slightly underestimates cortical thickness for cortices thinner than 1.0 mm (mean bias of −0.15 mm), similar to the values observed for the sternum in this study. For cortices between 1.0 and 3.0 mm, the method instead slightly overestimates thickness (mean bias +0.12 mm). These systematic tendencies should be considered when interpreting absolute thickness values. In addition, several Stradview parameters—such as normal length, smoothing, and surface fitting—require manual tuning to achieve stable results. These settings can influence the final thickness estimates, particularly in regions with complex geometry or low image contrast. Despite these limitations, CBM v2 remains the state-of-the-art method for estimating cortical thickness in thin cortices using clinical CT data. Accurate validation would require either high-resolution micro-CT imaging or direct histological measurements.

An additional factor potentially affecting cortical thickness estimation is the presence of costal cartilage calcification, which increases with age (Lau et al., 2015), at a rate of approximately 7% per decade (Holcombe et al., 2017). Calcified cartilage can appear as bone in CT images despite lacking true cortical structure. As a result, both the segmentation algorithm and the cortical bone mapping may misclassify calcified regions as cortical bone, particularly near the sternocostal junctions. This may lead to local overestimations of cortical thickness or irregularities in the segmented surfaces. While these effects are anatomically expected and largely confined to older individuals (as seen in Figure 8), they introduce an additional source of uncertainty that should be considered when interpreting regional thickness patterns.

A key limitation is that the cortical bone mapping approach has not been specifically validated for the sternum. While validated for long bones such as the femur, the sternum differs substantially in geometry, cortical thickness, and surrounding anatomical structures. This introduces uncertainty in the absolute thickness estimates and may affect both the PCA results and the derived biological interpretations. Consequently, the cortical thickness results should be interpreted with caution, particularly in a quantitative sense.

### Principal component analysis

In the present study, combining shape and cortical thickness within a single statistical shape model resulted in the first principal component primarily describing variation in cortical thickness. This contrasts with traditional shape-only SSMs, where the first PC typically captures overall size variation (Dryden and Mardia, 2016). When metrics with different magnitudes and spatial characteristics are combined, the ordering of PCs becomes sensitive to the chosen normalization strategy. Several normalization approaches were evaluated, and the selected method was chosen to preserve the geometric shape among spatial coordinates while allowing cortical thickness to contribute meaningfully to the variance structure. The relatively low variance explained by each PC is expected, as cortical thickness varies more rapidly between adjacent nodes than spatial coordinates and exhibits less consistent patterns across subjects. The results also indicate a weak correlation between shape and cortical thickness, suggesting that these two aspects of sternal morphology vary somewhat independently.

The average cortical thickness distribution revealed that the thickest regions were located along the sternocostal grooves. This may reflect mechanical loading from the costal cartilages during respiration or the presence of calcified cartilage at the sternocostal junctions, both of which will increase the apparent cortical thickness in CT-based measurements. The manubrium exhibited a pronounced anterior ridge along the superior–inferior axis, corresponding to the attachment region of the pectoralis major. The sternal body showed a largely homogeneous cortical thickness distribution on both anterior and posterior surfaces, with greater intersubject variability in the inferior anterior region.

The mean sternum exhibited a high degree of bilateral symmetry in both shape and cortical thickness, despite most individual subjects exhibiting some degree of asymmetry. This suggests that while asymmetry is common at the individual level, it does not follow a consistent directional pattern across the population. As a result, asymmetries cancel out when averaged, indicating that they are likely random rather than systematic features of sternal morphology.

### Linear regression model

The linear regression models showed generally weak associations between the demographic predictors and the PC scores, as reflected by the low $R^{2}$ -values for spatial coordinates and cortical thickness. This can be compared to the $R^{2}$ -value reported in similar studies: 0.29 for the pelvis shape (Brynskog et al., 2021), 0.74–0.77 for femur shape, and 0.31–0.36 for femur cortical thickness (Klein et al., 2015). Although the inclusion of first-order interaction terms increased the likelihood of identifying significant models for individual PCs, these terms also introduce a risk of overfitting due to the relatively small sample size and reduce the interpretability of the regression coefficients. The use of 10-fold cross-validation helped mitigate overfitting, and the subsequent predictor-isolation analyses provided a more intuitive understanding of how sex, age, stature, and BMI influence sternal morphology. To increase the complexity further, non-linear terms could also have been investigated. The limits in predictive power make this study primarily descriptive rather than predictive.

Conditioning on stature, BMI, and age, the predicted male sternal body was larger than the female sternal body, consistent with previous findings (Weaver et al., 2014; Jit et al., 1980). Interestingly, the manubrium was similar in size between sexes, as shown in Figure 7, resulting in a proportionally larger manubrium in females. The manubrial shape also differed between sexes: females exhibited a longer and narrower manubrium, whereas males showed a wider manubrium, particularly at the attachment of the first costal cartilage, in line with (Selthofer et al., 2006). When isolating stature, increased stature was associated with a longer sternum, although the effect was less pronounced than the sex effect. Together, these findings suggest that sternal size and shape may be sufficient for sex estimation in fields such as archaeology and forensics, consistent with earlier studies showing that sternal morphology can be used to distinguish between sexes (Singh et al., 2025).

Age-related effects included widening of both the manubrium and the inferior sternal body, as well as increased cortical thickness at the sternocostal junctions. The latter is potentially influenced by age-related calcification of the costal cartilage, which can appear as cortical bone in CT-based measurements. Higher BMI was associated with increased cortical thickness, consistent with observations in load-bearing bones (Marieb, 2001). Although the sternum is not a primary load-bearing structure, one possible explanation is that increased soft tissue mass alters respiratory loading patterns, potentially contributing to cortical adaptation. All these trends highlight that demographic factors influence sternal morphology in distinct ways, even if the overall predictive power of the regression models remains modest.

### Other limitations

The sample size in this study is at the smaller end of similar studies, where (Holcombe et al., 2024) included 250 subjects, (Brynskog et al., 2021), included 132 subjects, and (Klein et al., 2015) included 98 subjects. The limited number of 114 subjects in this study can affect the results, as outliers will more highly affect both the PCA and the linear regression. The data also lacks some elderly individuals, resulting in an uneven age distribution. This limits the generalization of the results.

A key limitation of this study is the exclusion of the xiphoid process. This structure was omitted because it is rarely fractured (Şimşek et al., 2022) and exhibits substantial anatomical variability, including complete absence in a small proportion of individuals (Vatzia et al., 2021). Such variability makes it difficult to incorporate the xiphoid into a morphometric model that relies on consistent anatomical correspondence across subjects. Although several studies have described the morphological variations of the xiphoid process (Akin et al., 2011; Babinski et al., 2015; Xie et al., 2014), little is known about how these variations relate to demographic factors. Also, sternums with foramen were excluded, which would have influenced the structural response. This is something that the current model misses to capture.

Another limitation concerns the demographic composition of the dataset and its potential selection biases. The sample represents an American population from New Mexico, predominantly consisting of Caucasian individuals, with smaller proportions of Native American, Hispanic, and African American subjects. In addition, the use of post-mortem CT data introduces inherent biases related to cadaveric sampling, such as potential non-representative health conditions and post-mortem selection effects. As sternal morphology may vary across populations, these factors limit the generalizability of the findings to other ethnic groups and to the broader living population. This should be considered when comparing the present results with studies based on different or unreported populations, as population-specific anatomical differences and sampling biases may influence both shape and cortical thickness.

## Conclusion

This study provides a detailed descriptive framework of sternal shape and cortical thickness variation. The weak correlations between demographic predictors and the PC-based regression models indicate that both shape and cortical thickness vary substantially at the individual level. Nevertheless, several consistent trends were identified: sex had a stronger influence on overall sternal size than stature, sternal width increased with age, and individuals with higher BMI exhibited slightly thicker cortices. Together, these findings highlight the complex interplay between demographic factors and sternal morphology and provide a foundation for future applications in biomechanics, clinical assessment, and population studies. Furthermore, knowledge of sternal morphology in human body modeling can enhance the accuracy of thoracic responses in crash simulation, thereby increasing the likelihood of developing restraint systems suitable for a broader segment of the population.

## Data Availability

The raw data supporting the conclusions of this article will be made available by the authors, without undue reservation.
