# Supplementary material for: A statistical shape model of the human sternum including cortical bone thickness
Source: Front Bioeng Biotechnol. 2026 Aug 7;14:1872352. doi: 10.3389/fbioe.2026.1872352 (PMC13493582; doi:10.3389/fbioe.2026.1872352)
Supplement: Supplementary file 1 [file Table1.docx]

| **Parameter** | **Value** |
| --- | --- |
| **Resolution** | Very High |
| **Smoothing (surface)** | High |
| **Line Length** | 12 mm |
| **Outer Density** | 100 HU |
| **Map Direction** | Optimal |
| **Smoothing (thickness)** | 2/20 |
| **Fill Missing Data** | 58-75% |
| **Reject Angles Above** | 25-50º |
