# Supplementary material for: A statistical shape model of the human sternum including cortical bone thickness
Source: Front Bioeng Biotechnol. 2026 Aug 7;14:1872352. doi: 10.3389/fbioe.2026.1872352 (PMC13493582; doi:10.3389/fbioe.2026.1872352)
Supplement: Supplementary file 2 [file Table2.docx]

| **PC number** | **Variation explained** | $\boldsymbol{R}^{\boldsymbol{2}}$ | Age | Stature | Sex | BMI | Sex:Stature | Sex:BMI | Sex:Age | Age:BMI | Age:Stature | Stature:BMI |
| --- | --- | --- | --- | --- | --- | --- | --- | --- | --- | --- | --- | --- |
| PC 1 | 20,82% | 0,26 | X | X | X | X |  |  | X |  | X | X |
| PC 2 | 7,43% | 0,70 |  | X | X |  |  |  |  |  |  |  |
| PC 3 | 4,77% | 0,16 | X |  | X |  |  |  | X |  |  |  |
| PC 4 | 4,12% | 0,31 | X |  |  |  |  |  |  |  |  |  |
| PC 7 | 2,26% | 0,20 | X | X | X | X |  |  |  | X |  |  |
